# Increased miR-7641 Levels in Peritoneal Hyalinizing Vasculopathy in Long-Term Peritoneal Dialysis Patients

**DOI:** 10.3390/ijms21165824

**Published:** 2020-08-13

**Authors:** Raquel Díaz, Pilar Sandoval, Raul R. Rodrigues-Diez, Gloria del Peso, José A Jiménez-Heffernan, Ricardo Ramos-Ruíz, Carlos Llorens, Gustavo Laham, Mabel Alvarez-Quiroga, Manuel López-Cabrera, Marta Ruiz-Ortega, María A. Bajo, Rafael Selgas

**Affiliations:** 1 Research Institute of La Paz (IdiPAZ), University Hospital La Paz, 28046 Madrid, Spain; rakadm@hotmail.com (R.D.); gloria.delpeso@salud.madrid.org (G.d.P.); mauxiliadora.bajo@salud.madrid.org (M.A.B.); rafael.selgas@salud.madrid.org (R.S.); 2 Red de Investigación Renal (REDINREN), Instituto de Salud Carlos III, 28029 Madrid, Spain; MRuizO@quironsalud.es; 3 Centro de Biología Molecular “Severo Ochoa” (CBM), Spanish Council for Scientific Research (CSIC), Universidad Autónoma de Madrid (UAM), 28049 Madrid, Spain; pilarsandovalcorrea@hotmail.com (P.S.); mlcabrera@cbm.csic.es (M.L.-C.); 4 Cellular and Molecular Biology in Renal and Vascular Pathology Laboratory, Fundación Instituto de Investigación Sanitaria-Fundación Jiménez Díaz, Universidad Autónoma Madrid, 28040 Madrid, Spain; 5 ISRIN (Instituto Reina Sofia de Investigación Nefrológica), 28003 Madrid, Spain; 6 Departamento de Anatomía Patológica, Instituto de Investigación Sanitaria La Princesa (IP), Hospital Universitario La Princesa, 28006 Madrid, Spain; jheffernan@yahoo.com; 7 Fundación Parque Científico de Madrid, Genomics Unit, 28049 Madrid, Spain; ricardo.ramos@fpcm.es; 8 BiotechVana S.L. Parc Cientific, 46980 Valencia, Spain; carlos.llorens@biotechvana.com; 9 Sección Nefrología del Centro de Educación Médica e Investigaciones Clínicas (CEMIC), C1431FWO Buenos Aires, Argentina; guslaham@yahoo.com.ar (G.L.); malvaq@gmail.com (M.A.-Q.); 10 Programa de Diálisis Peritoneal, Fresenius Medical Care Argentina, C1061AAA Buenos Aires, Argentina

**Keywords:** miRNAs, peritoneal dialysis, hyalinizing vasculopathy, peritoneum, kidney, endothelial-to-mesenchymal transition

## Abstract

Peritoneal hyalinizing vasculopathy (PHV) represents the cornerstone of long-term peritoneal dialysis (PD), and especially characterizes patients associated with encapsulating peritoneal sclerosis. However, the mechanisms of PHV development remain unknown. A cross sectional study was performed in 100 non-selected peritoneal biopsies of PD patients. Clinical data were collected and lesions were evaluated by immunohistochemistry. In selected biopsies a microRNA (miRNA)-sequencing analysis was performed. Only fifteen patients (15%) showed PHV at different degrees. PHV prevalence was significantly lower among patients using PD fluids containing low glucose degradation products (GDP) (5.9% vs. 24.5%), angiotensin converting enzyme inhibitors (ACEIs) (7.5% vs. 23.4%), statins (6.5% vs. 22.6%) or presenting residual renal function, suggesting the existence of several PHV protective factors. Peritoneal biopsies from PHV samples showed loss of endothelial markers and induction of mesenchymal proteins, associated with collagen IV accumulation and wide reduplication of the basement membrane. Moreover, co-expression of endothelial and mesenchymal markers, as well as TGF-β1/Smad3 signaling activation were found in PHV biopsies. These findings suggest that an endothelial-to-mesenchymal transition (EndMT) process was taking place. Additionally, significantly higher levels of miR-7641 were observed in severe PHV compared to non-PHV peritoneal biopsies. Peritoneal damage by GDPs induce miRNA deregulation and an EndMT process in submesothelial vessels, which could contribute to collagen IV accumulation and PHV.

## 1. Introduction

Nowadays peritoneal dialysis (PD) is one of the most convenient replacement treatments for end-stage renal disease patients (ESRD) [1]. However, the repeated exposure of the peritoneum to PD fluids (PDFs) evokes several cellular responses in the peritoneal membrane, including activation of an inflammatory response, angiogenesis and fibrosis, leading to a functional membrane failure that limits PD performance [2]. These alterations remain specially marked by the reiterated presence of unsolved peritonitis episodes in some PD patients [3,4,5]. Most of the preclinical studies, based on in vitro studies in mesothelial cells (MCs) and in animal models of PDFs exposure, have investigated the mechanisms involved in the peritoneal membrane damage [6]. These studies have demonstrated that MCs acquire myofibroblast characteristics through an epithelial-to-mesenchymal transition (EMT) process, known as mesothelial-to-mesenchymal transition (MMT) [7]. Myofibroblast conversion of MCs contributes to peritoneal fibrosis in PD patients [8,9,10]. Myofibroblasts are the main collagen-producing cells responsible for excessive accumulation of extracellular matrix proteins (EMC) found in fibrotic disorders [11]. Therefore, many studies have proposed that MMT is an important process in peritoneal fibrosis and therefore, targeting MMT could prevent peritoneal membrane functionality failure [12].

One remarkable lesion observed in PD patients is peritoneal hyalinizing vasculopathy (PHV), which associates with the end-stage peritoneal state (encapsulating peritoneal sclerosis). Although the histological characterization of PHV is well-known [13,14], its pathogenesis remains unclear due, in part, to the absence of an experimental model resembling human characteristics. PHV is characterized by homogeneous vascular wall hyalinization due to endothelial basement membrane reduplication that produces progressive vascular lumen occlusion [15,16]. These changes modify transcapillary ultrafiltration and limit the water and solutes exchange between blood and dialysate compartments. Ultrafiltration depends on the osmotic/hydrostatic pressure gradient and on the number of perfused intact peritoneal microvessels. Therefore, some authors have proposed the presence of vasculopathy in the peritoneal microcirculation as the main cause of ultrafiltration failure [17]. PHV is also accompanied by an increase in the capillary permeability, leading to the entry of plasma proteins and other soluble factors into the vascular wall, therefore promoting inappropriate inflammatory reactions. The use of PDFs with low-GDPs (glucose degradation products) has been associated with a lower incidence and severity [14], although exceptions appear among children [18].

Under pathological situations, endothelial cells can undergo phenotype changes leading to the activation of myofibroblasts, in a process named endothelial-to-mesenchymal transition (EndMT) [19]. Many studies have demonstrated that EndMT is an important mechanism in myofibroblasts generation in experimental fibrosis [20,21] and in some human fibrotic disorders [19,22,23,24]. Although a wide range of factors and pathways have been involved in fibrosis-associated EndMT, microRNAs (miRNAs) have recently suggested as the main factors implicated in its pathogenesis [25,26,27]. MiRNAs are evolutionarily conserved small (20–24 nt) non-coding RNAs involved in both stability and translation of target mRNAs. Since their discovery, miRNAs have progressively moved to a central stage in the understanding of the post-transcriptional gene expression regulation. The importance of miRNAs function has been demonstrated in a wide variety of processes including diabetes, cancer, cardiovascular and renal diseases, and peritoneal fibrosis [28,29,30,31,32,33,34].

Herein, we have investigated the incidence of PHV in a cohort of patients under PD treatment, defining their risk or protective factors and investigating potential molecular mechanisms involved in the pathogenesis of vascular lesions. We hypothesize that glucose and especially GDPs from PDFs could promote endothelial phenotype changes leading to EndMT, therefore contributing to an aberrant perivascular accumulation of collagen IV. To find potential therapeutic options for this clinical problem, the effect of different treatments of PD patients on PHV lesions was also evaluated. Finally, to search for novel therapeutic strategies in PHV a miRNA-sequencing experiment was designed, to find a miRNA candidate to be used as a molecular target.

## 2. Results

### 2.1. Clinical Characteristics of Patients

**Figure 1:**
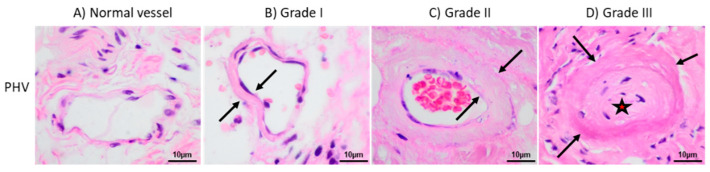
Images of the different grades of peritoneal hyalinizing vasculopathy (PHV). (**A**) Normal vessel showing no thickening. (**B**) Grade I is characterized by minimal thickening (area between arrows) with no luminal obliteration. (**C**) In grade II lesions, the thickening is moderate (area between arrows) and luminal narrowing is present. (**D**) Grade III lesion showing intense thickening (arrows indicate vascular external limit) with trapped cells and complete luminal obliteration (red star).

**Figure 2:**
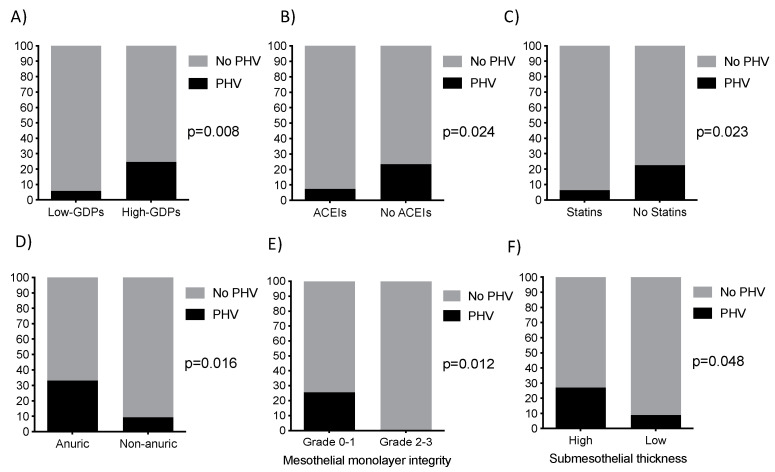
Prevalence of PHV was significant lower in patients using (**A**) biocompatible peritoneal dialysis (PD) solutions, (**B**) angiotensin converting enzyme inhibitors (ACEIs) and (**C**) statins. Patients with preserved residual renal function (**D**), peritoneal mesothelial integrity (**E**) and lower peritoneal fibrosis (**F**) also showed significantly lower PHV prevalence.

Clinical characteristics of the Spanish patients under PD with and without PHV lesions are shown in Table 1. Peritoneal biopsies samples were obtained during transplant surgery in 84% of patients, and the remaining samples were collected during incidental abdominal wall surgery. No patient had membrane failure at the time of biopsy. The most frequent ESRD cause was glomerulonephritis, followed by polycystic disease and diabetes. The PHV presence was not related to kidney disease etiology. A total of fifteen patients (15%) showed PHV at different histological degrees (eight patients presented grade I, six patients grade II and one patient grade III) (Figure 1). The PHV prevalence was significantly lower in patients receiving low-GDP PDFs than those treated with high-GDP PDFs (5.9% vs. 24.5%, *p* = 0.008) (Figure 2A). Similarly, patients treated with angiotensin converting enzyme inhibitors (ACEIs) (7.5% vs. 23.4%, *p* = 0.024) or statins (6.5% vs. 22.6%, *p* = 0.023) showed lower PHV prevalence (Figure 2B,C). Residual renal function, showed to be also a protective factor for PHV (0% in patients with preserved renal function vs. 25.5% in anuric patients, *p* = 0.012) (Figure 2D). In addition, a multivariate logistic regression analysis showed that the factors associated with lower presence of PHV were ACEIs treatment (OR 0.15; 95% CI 0.03–0.65; *p* = 0.011), statins treatment (OR 0.17; 95% CI 0.03–0.87; *p* = 0.034), low-GDP PDFs (OR 0.18; 95% CI 0.03-0.86; *p* = 0.032) and preservation of residual renal function (OR 0.65; 95% CI 0.49–0.85; *p* = 0.002). Mesothelial monolayer integrity was associated with PHV absence (25.5% in grade 0–1 vs. 0% in grade 2–3, *p* = 0.012), whereas PHV lesions were correlated to submesothelial fibrosis (27% in higher vs. 8.8% in lower submesothelial thickness) (*p* = 0.048) (Figure 2E,F).

### 2.2. Aberrant Perivascular Accumulation of Collagen IV in Peritoneal Hyalinizing Vasculopathy

**Figure 3:**
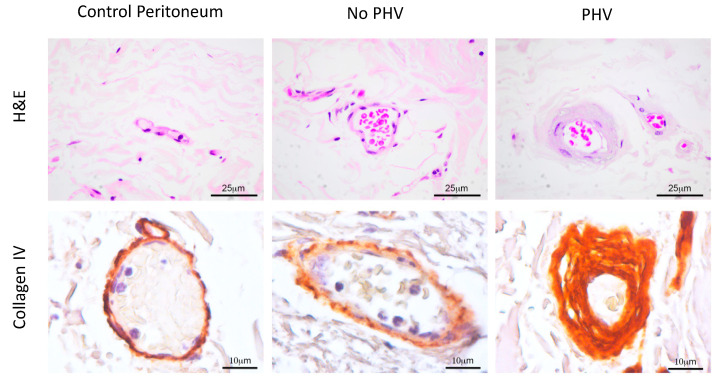
Collagen IV immunohistochemical analysis in peritoneal biopsies. A representative hematoxylin–eosin (H&E) image shows subendothelial thicknesses of the basement membrane in a PHV lesion. Immunostaining images show a PHV strongly stained for collagen IV in the basement membrane, as compared to a control sample or a PD-treated patient without PHV.

Biopsies from parietal peritoneum of patients showing PHV-related histological changes were analyzed for collagen IV, as compared to healthy peritoneum samples and PD-treated patients with apparently normal vessels in the submesothelial compact zone. Control and non-PHV biopsies demonstrated collagen IV staining limited to a thin circumferential area that corresponds to the normal endothelial basement membrane. However, samples showing PHV showed some vessels with uniform circumferential thickening of the vascular walls that correlated with an intense collagen IV accumulation (Figure 3).

### 2.3. Endothelial-to-Mesenchymal Transition in Peritoneal Hyalinizing Vasculopathy

**Figure 4:**
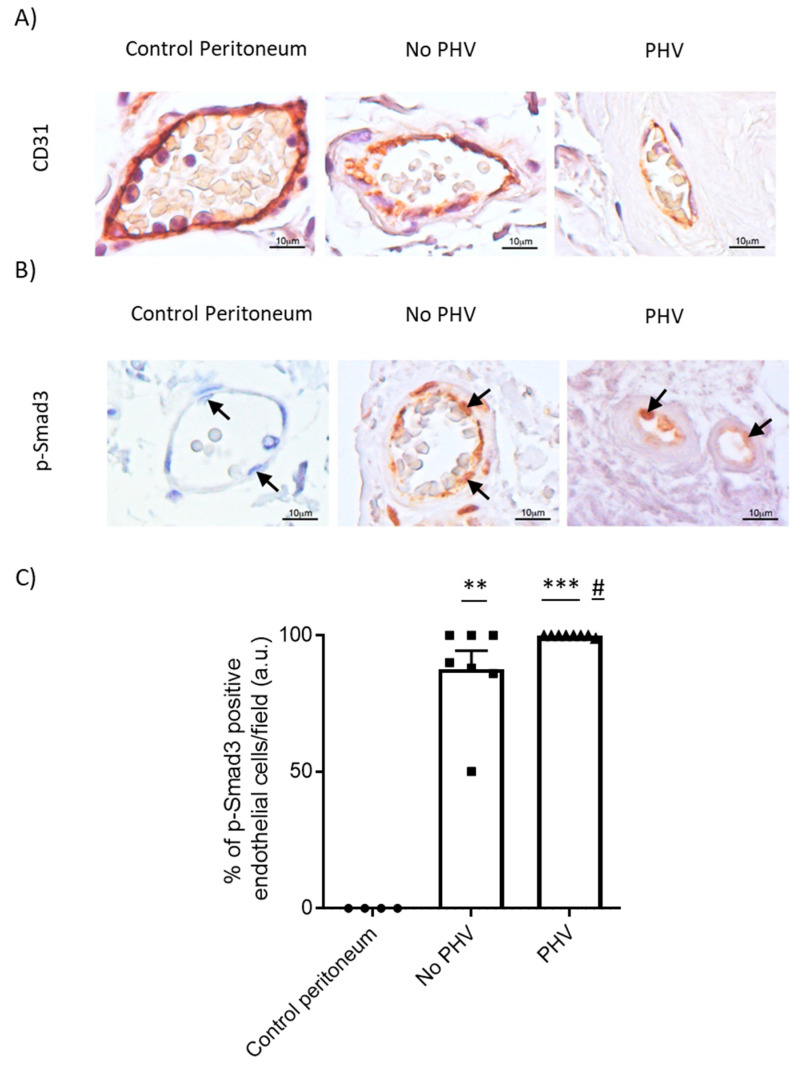
Immunohistochemical staining for CD31 and p-Smad3. (**A**) Endothelium of control peritoneal samples and PD-treated biopsies without PHV lesions present strong staining for CD31. On the contrary, endothelial cells of biopsies with PHV showed low intensity staining for CD31. (**B**) Endothelial cells from healthy biopsies were p-Smad3-negative. Nuclear p-Smad3 staining was found in the peritoneal endothelium of capillaries from PD-treated patients without and with PHV. Arrows point to endothelial cells. (**C**) pSmad3-positive endothelial nuclei were quantified and expressed as percentage of endothelial cells showing p-Smad3 positive staining per field. Bar graphic represents the mean ± SEM. ** *p* < 0.01 vs. control group; *** *p* < 0.005 vs. control group; # *p* < 0.05 vs. no PHV group.

**Figure 5:**
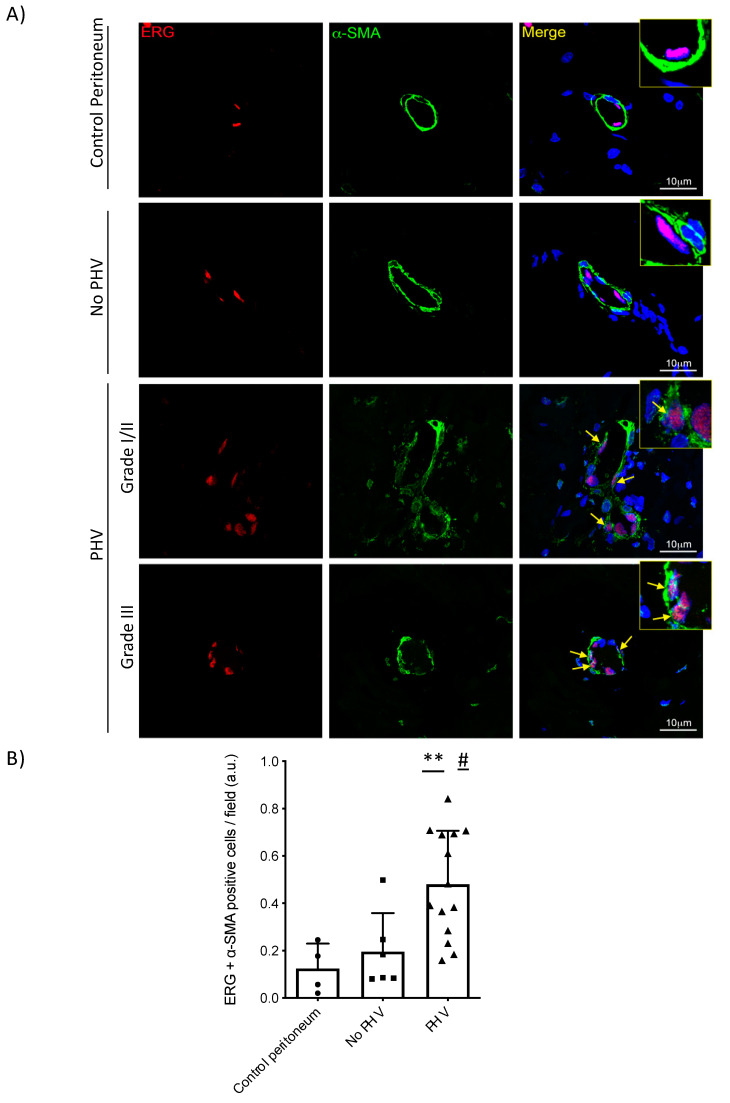
Detection of endothelial-to-mesenchymal transition in biopsies showing PHV. (**A**) Dual immunofluorescence staining representative pictures for ERG (endothelial marker) (red) and α-SMA (myofibroblast marker) (green) in a peritoneal capillary from a control sample, a PD-treated patient without PHV and patients presenting PHV at different degrees. Arrows point to double positive (ERG + α-SMA) endothelial cells indicating EndMT. Of note, perivascular smooth muscle cells are strongly marked for α-SMA in a normal vessel and in a PD patient without PHV. Perivascular smooth muscle cells seem to disappear in high-grade PHV biopsies. Insets show higher magnification of the delimited areas. (**B**) Double positive (ERG + α-SMA) endothelial cells were quantified. Graphic represents individual data points and the mean ± SEM. ** *p* < 0.01 vs. control group; # < 0.05 vs. No PHV group.

To test whether EndMT could be involved in PHV, the endothelial cell adhesion molecule CD31 [35] was investigated. Interestingly, immunohistochemistry revealed a relative decrease in the intensity of CD31 staining in the endothelium of PHV biopsies, as compared to control and PD-treated patients who did not develop submesothelial arteriolopathy (Figure 4A) which was also confirmed by immunofluorescence (Figure S1A). Alternatively, fibroblast-specific protein-1 (FSP-1) staining was observed in PHV endothelial cells, which helped to support an EndMT process happening in the peritoneal vessels of these patients (Figure S1B). Accordingly, we have detected a significant nuclear staining of phospho-Smad3, the TGF-β1 downstream effector, in the endothelium of patients undergoing PD, in both no PHV (*p* = 0.006) and PHV (*p* = 0.002), as compared to control peritoneal arterioles where endothelial cells were mainly negative (Figure 4B). This result suggests an activation of the TGF-β1-Smad3 pathway in the endothelium of the PDF-exposed microvasculature, which was further significantly maintained during PHV development (*p* = 0.04). In order to correlate both PHV-associated alterations (lack of the intercellular junctions and TGF-β-Smad3 signaling activation) with a potential initiation of an EndMT process, we performed a dual-immunofluorescence staining using erythroblast transformation-specific (ETS)-related gene (ERG) as endothelial marker and alpha-smooth muscle actin (α-SMA) as indicator of myofibroblast conversion. A significant increase in the percentage of double-positive endothelial cells in patients showing PHV signs, as compared to control (*p* = 0.007) and PD-exposed capillaries without histological alteration (*p* = 0.01) suggested the initiation of an EndMT process associated twith PD-induced PHV (Figure 5).

### 2.4. MicroRNA-Sequencing and qRT-PCR Results

**Figure 6:**
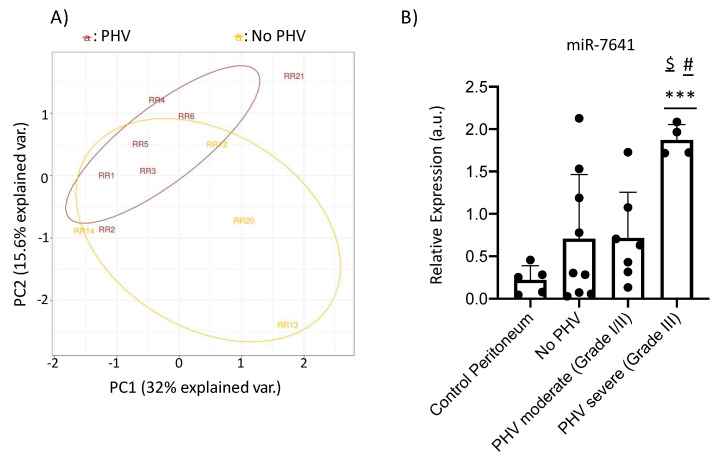
(**A**) Principal component analysis showing dissociation between PHV and no PHV groups. (**B**) Relative miR-7641 expression between samples. Data are represented as 2E(-dCt) as a measure of the relative amount of miR764. Graphic represents individual data points and the mean ± SEM. *** *p* < 0.001 vs. Control peritoneum, # *p* < 0.01 vs. No PHV group; $ *p* < 0.005 vs. PHV moderate group.

A number of 19 samples (11 PHV and 8 no PHV) were selected to perform small-RNA sequencing. After removal of samples showing poor quality we could analyze and compare 11 samples (seven PHV vs. four no PHV). We detected the expression of 1666 molecules, 706 out of which were recognized as miRNA. The principal component analysis (PCA) based on Fragments Per Kilobase Million (FPKM) data detected a clear dissociation between groups (Figure 6A). Therefore, small-RNA expression seems to be a differential characteristic between both groups of patients. Differential expression analysis (Cufflinks) suggested that up to 24 miRNAs might be regulated in PHV as compared to no PHV (Table 2). However, due to the special difficulties associated with these samples, which have been formalin fixed and show low cellularity, together to the low number of samples that we could analyze, we set out to validate their expression using an alternative technique of real time RT-PCR and using further series of samples. To perform this study, we included five control peritoneal biopsies, three additional PHV and no PHV patients as well as a second series of patients (six PHV and four no PHV). The new patient series included high-degree PHV samples (grade III) so we could split our samples in different categories according to the PHV severity. When available, RNA derived from the first series was also used for confirmation purposes (two PHV and two no PHV). Therefore, a total of five controls, nine PHV (three of them of grade III) and 11 no PHV were finally analyzed.

All selected miRNAs were analyzed by RT-qPCR. Some of the miRNAs were not detected in significant amounts while others did not show reproducible or statistical differences with the larger cohort of samples and were discarded. Among all studied miRNAs, only miR-7641 seemed to be consistent in the selected groups of patients. Our data showed a differential expression of miR-7641 between severe PHV (Grade III) patients as compared to both control (*p* < 0.001) and no PHV (*p* = 0.012). Similarly, there was a significant increase in miR-7641 levels in severe PHV compared to moderate PHV (Grade I/II) (*p* = 0.003). Although miR-7641 levels were also slightly enhanced in moderate PHV patients compared to No PHV (median of No-PHV = 0.303 a.u.; median of moderate PHV = 0.631 a.u.), in this case the difference did not reach statistical significance (Figure 6B). As a result, our data support that miR-7641 is a microRNA candidate to participate in PHV generation and/or evolution in patients under PD treatment.

## 3. Discussion

The main finding of this study is the description of EndMT as a major mechanism involved in PHV. The exposure of the peritoneal membrane to high-GDP PDFs, apart from causing basement membrane reduplication, can induce an endothelial phenotypic change into a myofibroblast-like cell via Smad3 activation, leading to an aberrant collagen IV accumulation in the peritoneal vascular wall. These processes could contribute to PHV, vessel lumen occlusion and subsequently membrane dysfunction with ultrafiltration failure.

The microvasculature of parietal peritoneum is mainly located between the submesothelial interstitial layer and peritoneal adipose tissue [36]. Long-term PDF exposure causes submesothelial fibrosis and, in some cases, encapsulating peritoneal sclerosis (EPS) and/or PHV. The PHV histology is defined as hyalinization of capillary or post-capillary venule walls, reduplication of the vascular basement membrane and narrowing or obstruction of the lumen [15]. However, the cause of PHV is undetermined. Here, we consider a large series of non-selected PD patient biopsies, leading us to show a strong association between high-GDP PDFs and PHV events. These data confirm previous observations from our group, where biocompatible low-GDP PDFs preserved the mesothelial monolayer and vessel wall integrity better than conventional ones [14]. Accordingly, peritoneal morphological changes, including PHV, are aggravated with long-term PD [17,37,38]. Interestingly, microvascular system alterations of diabetes mellitus patients were associated with increased type IV collagen biosynthesis, both at gene and protein level, and posttranslational alterations were detected, especially glycation, leading to advanced glycation end-products (AGEs) and collagen covalent crosslinks. These collagen alterations decrease collagen extractability and collagenases susceptibility, favoring glomerular basement membrane thickening [39,40]. Interestingly, AGEs deposition has also been observed to be especially intense in the vessel wall of long-term PD patients showing PHV [15]. According to previous studies [41] our data show an aberrant detection of subendothelial type IV collagen in pathological peritoneal biopsies, suggesting PD-related factors, including high-GDP PDFs and AGEs deposition in the vascular wall, as an important candidate mechanism implicated in PHV pathogenesis. This result could be paradoxical, taking into account previous studies indicating loss in collagen IV synthesis and fibrillar collagen I and collagen III expression when EndMT is present [42]. Collagen IV is characteristic of basement membranes, and it is produced by endothelial cells and, most likely, by pericytes. As commented, the peculiarity of PHV is the wide reduplication of the basement membrane induced by high glucose and their degradation products, which could explain why an increase in the basement membrane thickening, high collagen IV levels and EndMT process are present together. In this context, our previous observation regarding the absence of hyalinizing vasculopathy outside the peritoneal cavity in patients undergoing PD demonstrates that the main contributors for PHV severity are local PD-related factors. We cannot exclude a contribution of uremia in the development of the lesion but it does not seem responsible for its intensity [43].

In the context of the PHV, the general absence of cellular entities surrounding microangiopathies, lead us to focus on the endothelial cell as the principal responsible for the morphological alterations observed. Experimental mouse models resembling cardiovascular diseases and organ fibrosis have shown that endothelial cell injury can involve phenotype changes toward the acquisition of mesenchymal or myofibroblastic characteristics and therefore, loss of the original functional properties, via an EndMT process [19,44]. Importantly, a recent study of single-cell RNA-sequencing of renal endothelium under hyperosmotic stress conditions revealed a great heterogeneity between endothelial cells from different locations, remarking the importance of considering the intrinsic tissue-specific characteristics, as well as disease-specific studies [45]. In human peritoneal biopsies, we have noted a CD31 staining decrease in capillary lesions as compared to those cases showing an apparent conservation of the vessel wall integrity. In fact, the function of the vascular cell adhesion and signaling molecule, CD31, in endothelial cells, has been related to vascular permeability barrier preservation. At the same time, CD31 supports the integrity of endothelial cell–cell junctions and provide protection from the microvascular network [35]. While not clearly documented, it is tempting to speculate that many of the molecular and phenotypic changes described for other transitional programs (e.g., EMT or MMT), could also play critical roles in orchestrating an EndMT process [46]. In this aspect, the loss of endothelial markers, such CD31 and vascular endothelial cadherin (VE-Cadherin), as well as the acquisition of fibroblast- or myofibroblast-specific markers such as FSP-1, α-SMA, collagens and vimentin during EndMT processes is accepted [47]. Here, we observed loss of CD31 and gain of FSP-1 expression, as well as the presence of capillary endothelial cells (ERG+) co-expressing the myofibroblast marker α-SMA in PHV lesions. It is important to highlight that in control biopsies, as well as in cases not presenting PHV, there was no α-SMA staining other than that observed in the smooth muscle cells surrounding the endothelium. In fact, electron microscopy revealed degenerative changes of vascular smooth muscle cells in the peritoneal walls in PHV cases [41]. The lack of single α-SMA positive smooth muscle cells associated with arteriolopathies lead us to discard a specific role for these cells during the EndMT process. Although a previous study performed in a mouse model of chronic exposure of peritoneum to PDF suggested that a small percentage of myofibroblasts can be originated from endothelial cells via EndMT [48], currently there are no experimental models that resemble the PHV histopathological characteristics described in patients, which limits attaining a whole picture of the molecular mechanisms that govern the EndMT process, as well as precludes a deeper exploration into potential therapeutic targets.

Here, we have found a significant lower prevalence of PHV lesions in PD patients treated with ACEIs or statins. ACEIs act blocking the generation of Angiotensin II (Ang II), the effector peptide of the renin angiotensin system, and are indicated in patients on dialysis [49]. Ang II can elicit many molecular and cellular responses, including inflammation, angiogenesis, MMT and fibrosis, by the production of several mediators, such as TGF-β and VEGF [30,50,51], therefore contributing to peritoneal damage. Previous data have shown the protective effect of ACEI treatment in both, glucose-exposed cultured human peritoneal MCs and animal models of PDF exposure [50,52,53,54]. Moreover, Ang II induces phenotype conversion in many different cells, including endothelial cells [55,56]. In fact, Ang II-induced EndMT participates in cardiac fibrosis [56]. All these findings support the idea that the protective effects against PHV observed in PD patients under ACEI treatment could be due to Ang II blockade on endothelial cells. Statins besides cholesterol-lowering drugs also exert pleiotropic actions, as demonstrated in cardiovascular diseases [57]. In ESRD patients, statins diminished inflammatory-related parameters, although they do not improve overall survival, and some data indicate an association with greater progression of coronary artery calcification [58,59]. In addition, statin therapy has been correlated with improved survival in incident PD patients [60]. Studies in cultured peritoneal MCs under high glucose conditions showed that statins may have protective effects by inhibiting the production of growth factors and blocking MMT [61,62]. In preclinical models of PDF exposure in rats, atorvastatin treatment prevented alterations in peritoneal transport and diminished peritoneal thickness [63], and simvastatin restored MMT-related changes [62]. Therefore, we suggest statins as a promising therapeutic strategy to be considered in order to preserve the peritoneal membrane integrity and diminish the presence of PHV events in long-term PD patients. The multivariate analysis demonstrated the independence of this protective factor regarding to the use of high/low glucose degradation products. This finding also applies to ACEI use.

The complement system is an important mediator of the incompatibility reactions induced by PDF components, particularly glucose and GDPs [64]. A proteomics prospective study achieved in adult PD patients, identified complement components as possible EPS biomarkers [65]. Accordingly, proteomic profiling studies from children under PD treatment identified a total of 18 complement components in the peritoneal effluent [18]. Interestingly, the latter study, also detected high abundance of complement deposition, including C1q, C3d and terminal complement complex, in PD parietal arterioles, which was correlated with the TGF-β-Smad-dependent signaling, as well as with the severity of vasculopathy [18]. The well-described role of the complement activation system in the inflammatory response associated with the PD treatment, suggests that it could be a potential mechanism of peritoneal membrane damage [66]. On the other hand, the TGF-β-Smad-dependent signaling pathway is considered the main mechanism involved in MMT-related fibrotic peritoneal diseases [67,68], including PD [30]. In fact, numerous preclinical approaches, including TGF-β blocking peptides, Smad7 gene transfer and bone morphogenetic protein 7 treatment, have been demonstrated to protect the peritoneal membrane from dialysate-induced damage [48,69,70]. Here, we reveal a significant activation of the TGF-β-Smad3-dependent signaling pathway in the endothelium of patients receiving PD either with or without PHV lesions, as compared to healthy peritoneal samples. The Smad-dependent pathway in TGF-β family signaling participates in EndMT associated with renal, pulmonary or cardiac fibrosis [20,21,24,71]. However, our findings suggest, for the first time, a link between TGF-β-Smad3-dependent signaling and EndMT in the context of the PHV. Taking into account that Ang II can directly activate the Smad signaling system and shares with TGF-β many intracellular signals implicated in vascular fibrosis [72,73], the protective effect against PHV developing observed in our series of PD patients receiving ACEIs could be due to the blockade of Ang II/Smad activation on endothelial cells.

Increasing studies describe the importance of miRNA regulation to maintain peritoneal cavity homeostasis during PD [74]. In this regard, miR-9-5p inhibited myofibroblast conversion of effluent-isolated MCs from patients undergoing PD as a self-limiting homeostatic response [75]. Some authors highlight the pivotal role of miRNAs in the development and progression of peritoneal fibrosis, indicating that they may represent potential biomarkers and therapeutic options [33]. Although, most studies were performed in peritoneal effluents, serum of patients undergoing PD or in animal experimental models, one study developed in peritoneal membrane biopsies from pediatric patients, showed an increase in miR-21 levels associated with fibrotic stages [34]. Alternatively, studies using primary/immortalized and microvascular/aortic endothelial cells reported the participation of various miRNAs in the regulation of high glucose-induced EndMT [76,77,78]. However, the extrapolation of in vitro findings to human diseases using an extended diversity of endothelial cells should be carefully considered. At systemic level, some miRNAs have been proposed as potential connectors between sclerosis tissue fibrosis and vasculopathy alterations [25]. Here we developed a complete miRNA-sequencing profiling analysis in peritoneal membrane biopsies from adult patients under PD with and without PHV. It is important to remark the absence of a preclinical model to study PHV, that clearly limits the knowledge in this field. We found a total of 24 miRNAs dysregulated in peritoneal samples with PHV as compared to no PHV patients, including miRNA-34a-5p. Similarly, a miRNA profiling study in fibrotic rat peritoneal tissue identified an increased miRNA-34a-5p expression [79]. However, the low amount of RNA extracted from the formalin-fixed paraffin-embedded (FFPE) tissues, as well as the low cellularity that characterizes peritoneal samples only allowed us to confirm a significant upregulation of miR-7641 by RT-qPCR in biopsies showing PHV. miR-7641 is a poorly investigated miRNA expressed dominantly in different breast and colon cancer cell lines and it has been proposed as a promising targeting factor for cancer therapy due to its capacity to regulate ribosomal proteins [80]. Curiously, there is only one study concerning miR-7641, which describes its role during endothelial differentiation from embryonic stem cells [81], suggesting a potential role of this miRNA in the endothelial cell differentiation during the EndMT process associated with the PHV pathogenesis that we propose here.

In summary, this study reveals for the first time EndMT-related changes associated with the exposition of the submesothelial microvasculature to high-GDP solutions in long-term PD patients as a major mechanism involved in the pathogenesis of PHV. Although further studies testing the specific role of miR-7641 in the context of PHV are necessary due the lack of knowledge about this miRNA, there is an opportunity to shed light on mechanisms that govern peritoneal membrane damage in PD patients.

## 4. Materials and Methods

### 4.1. Patients and Peritoneal Samples

Formalin-fixed paraffin-embedded (FFPE) parietal peritoneal biopsies corresponding to a total of 100 PD patients were recovered from January 2001 to June 2019 in Hospital Universitario La Paz, Madrid, Spain. Variables related to their personal history, regular treatments and DP-related events are shown in Table 1. Samples were taken under patients’ informed consent for the opportunity of invasive surgical procedures (mainly kidney transplantation). The study protocol was approved by the local Bioethics Committee of Hospital Universitario La Paz, Madrid, Spain. In order to complete histological and miRNA-sequencing studies, an additional series of 6 biopsies showing PHV and 4 with no PHV was collected from the Hospital Universitario Centro de Educación Médica e Investigaciones Clínicas (CEMIC), Buenos Aires, Argentina. Informed written consent to use surgical samples was also obtained from patients and Helsinki declaration was considered. A total of 9 control parietal peritoneal membrane samples were obtained from no uremic PD-unrelated organ donors in Spain. Of them, 4 samples were used for immunohistochemical and dual-immunofluorescence studies and 5 for miRNA validation assay. All biopsies were fixed in neutral-buffered 3.7% formalin and embedded in paraffin in similar conditions [8]. After routine pathologic study, only those biopsies that showed an optimal conservation of the peritoneal structure were selected for the study.

### 4.2. Immunohistochemistry

Deparaffinized 3-µm sections were heated to expose the hidden antigens using Real Target Retrieval Solution containing citrate buffer, pH 6.0 (Dako, Glostrup, Denmark). Endogenous peroxidase was blocked with Real Peroxidase-Blocking Solution (Dako). Samples were stained using primary antibodies to detect collagen IV (monoclonal mouse, clone CIV 22, 1:50, Dako), CD31 (monoclonal mouse, clone JC70A, 1:20, Dako), *p*-Smad3 (monoclonal rabbit, clone C25A9, 1:300, Cell Signaling Technology, Danvers, MA, USA) or FSP-1 (S100A4, polyclonal rabbit, 1:800, Dako). A biotinylated goat anti-mouse or anti-rabbit IgG secondary antibody (Vector Laboratories, Burlingame, CA, USA) was applied to detect primary antibodies. Complexes were visualized using the R.T.U Vectastain Elite ABC Kit (Vector Laboratories) and 3,3′-diaminobenzidine (DAB) (Dako) as chromogen. Finally, tissue sections were counterstained with hematoxylin. Negative controls, in which primary antibodies were omitted, were used to verify the specificity of the technique. Almost five arbitrary fields (magnification 400×) for each sample were quantified for endothelial p-Smad3 staining which was expressed as percentage of endothelial cells showing p-Smad3-positive staining per field.

### 4.3. Morphological Parameters Analyzed

To evaluate the MC integrity a semi-quantitative scale (grade 3 = normal cell density; grade 0 = complete denudation) was used as described by Plum et al. [82]. Submesothelial thickness was classified as normal or Grade 1 (thickness of the compact zone less than 150 μm), moderate thickening or Grade 2 (150–350 μm) and intense thickening or Grade 3 (greater than 350 μm). The PHV classification was performed according to the scale described by Honda et al. [83]: grade I = mild thickening without stenosis of the lumen; grade II = moderate thickening with partial luminal stenosis; and grade III = intense thickening with marked stenosis and luminal distortion or complete occlusion.

### 4.4. Dual-Immunofluorescence

FFPE sections (3 μm) were deparaffinized and heated to expose the hidden antigens using Real Target Retrieval Solution (Dako). Samples were stained for immunofluorescence using primary antibodies to detect ERG (monoclonal rabbit, clone EPR3864, 1:800, Abcam, Cambridge, UK) and *α*-SMA (monoclonal mouse, clone 1A4, 1:3000, Sigma-Aldrich, St. Louis, MO, USA). Secondary antibodies Alexa 647 and Alexa 555 were incubated (Invitrogen, Carlsbad, CA, USA) at room temperature. Finally, the slides were mounted with 4,6-diamidino-2-phenylindole (DAPI) nuclear stain (Invitrogen, Carlsbad, CA, USA). Negative controls, in which primary antibodies were omitted, did not give rise to any detectable labeling. Images were captured with a LSM710 Zeiss Confocal Microscope (Zeiss, Oberkochen, Germany) and almost five arbitrary fields (magnification 630×) for each sample were quantified using the analysis software Image-J (National Institute of Health, Bethesda, MD, USA).

### 4.5. miRNA Extraction

miRNAs from paraffin-embedded samples were extracted using a commercial kit (RNeasy FFPE kit, Qiagen, Duesseldorf, Germany). Samples, tools, and working surface were cleaned out using RNaseZap (Invitrogen, Thermo Fisher Scientific, Waltham, MA, USA) to avoid RNAses. Total RNA was quantified using RiboGreen^®^-based fluorescent determination and analyzed in Bioanalyzer (Agilent Technologies, Cheadle, UK) to test size profiling, which mainly consisted in low molecular species as expected for FFPE samples, and confirmed concentration of RNA preparations, which typically reached around 5–20 ng/uL.

### 4.6. miRNA-Sequencing Analysis

Nineteen samples (11 PHV and 8 no PHV) were selected for sequencing of smallRNAs. Libraries were prepared according to the instructions of the “NEBNext Multiplex Small RNA Library Prep Set for Illumina” kit from New England Biolabs (New England BioLabs, Ltd., Ipswich, MA, USA). The input amount of total RNA to start the protocol was 30–120 ng of each sample, quantified by bioanalyzer using RNA 6000 Nano Chips and/or fluorimetry. The library preparation procedure included a PCR step, which was adjusted to 13 cycles. The libraries obtained were run in PAGE, gel purified and quantified using an Agilent 2100 Bioanalyzer with High-Sensitivity DNA chips. The pool of size-selected libraries was titrated by quantitative PCR using the “Kapa-SYBR FAST qPCR kit forLightCycler480” (KapaBioSystems, Wilmington, MA, USA) and a reference standard for quantification. This final pool of libraries was denatured prior to being seeded on a flow-cell at a density of 2.2 pM, where clusters were formed and sequenced using a “NextSeq™ 500 High Output Kit”, in a 1 × 75 single-end sequencing run on a NextSeq500 sequencer (Illumina). An amount of 10–25 million of single-end reads (mean 16.8 × 10^6^) was obtained per sample. The quality of sequences as viewed on fastq files was assessed using the program FastQC-0.11.7 (http://www.bioinformatics.bbsrc.ac.uk/projects/fastqc). Sequences were then filtered according to quality (mean Q value of 30), size (minimum length of 16) and ambiguities (removing reads with more than 10% Ns within the sequence). For that purpose, the program Prinseq [84] was used. Numbers of sequences per sample are summarized in Table S1. Next, sequences were mapped against human genome (release GRCh38.p10) using TopHat [85,86] and reads were annotated using the corresponding Hs38-gtf file. The whole bioinformatic protocol was executed using the RNAseq pipeline app of the GPRO-suite [87]. Eight samples showed significantly low mapping yields and were removed from the study (Table S1). Thus, a final number of 11 samples (7 PHV and 4 no PHV) averaging 16 million reads were used for differential expression analysis, made using Cufflinks/CuffDiff [85,86] to compare the expression of PHV vs. no PHV. We filtered out those entries whose detection levels were lower than 40 reads (sum of averages from PHV and no PHV groups) and finally found a number of 1666 molecules which were considered as positively detected.

### 4.7. miRNA Validation by Real Time-Quantitative PCR (RT-qPCR)

We selected 23 microRNAs as candidates to be analyzed in further validation studies (Table S2; note that a predesigned assay for miR-383.5p.2 was not available), as well as 4 miRNAs used as reference genes (selected according to their stable expression in the miRNAseq analysis). MicroRNA-specific reagents were obtained from Exiqon (LNA-modified RT-PCR primers, Bionova, Spain). Samples were reversed-transcribed (miRCURY RT kit) and miRNA expression was estimated by real time PCR, run in a qPCR LC480 equipment (Roche, Pleasanton, CA, USA). RT samples were spiked and analyzed for cel39 (Exiqon, Vedbaek, Denmark) to ensure the absence of inhibition of RT-PCR reactions. Samples (10 ng of total RNA) were measured in triplicate for each relevant miRNA strictly following manufacturer’s recommendations. For normalization, we used the average expression of endogenous miRNAs (miR-23a, miR-132 and miR-134) (miRNA miR-203 was not considered due to low detection among samples) to calculate deltaCt (dCt) values. Target microRNAs showing negligible or low expression levels in qPCR reactions were removed from the study, so only miRNAs showing medium to higher expression were analyzed. miRNAs which were removed at this level were miR-1185, miR-1193, miR-323b, miR-376a, miR-412, miR-494, miR-548, miR-6507 (5p,3p) and miR-873, and were not further investigated. Among the rest of the miRNAs, their differential expression was further analyzed using an additional series of samples (a cohort of patients from Argentina) in which we could categorize between medium and high PHV (grade III) patients. A table including deltaCt values (expressed in arbitrary units) for miR-7641 microRNA of all samples tested in the study is included as Table S3.

### 4.8. Statistics

Physiological values are expressed as percentages and mean ± standard deviation (SD). Percentages were compared using the Chi-square test and means with Student *t*-test or Mann–Whitney U-test (non-parametric data). Univariate and multivariate logistic regression analyses were employed to investigate the factors associated with the presence of PHV on peritoneal biopsy. RQ (Relative Quantification levels) from RT-qPCR studies were compared using one-way analysis of variance (ANOVA) and a Tukey post-hoc test. All those statistical analyses were completed using SPSS 14.5 (Chicago, IL, USA) or GraphPad Prism (La Jolla, CA, USA). Results are presented as the mean ± standard error of the mean (SEM) in graphics of Figure 4, Figure 5 and Figure 6. A *p* value < 0.05 was considered statistically significant.

### 4.9. Data Availability

Raw data have been deposited at the NCBI SRA archive with BioProject record PRJNA638799, and BioSample records SAMN15208325, SAMN15208326, SAMN15208327, SAMN15208328, SAMN15208329, SAMN15208330, SAMN15208331, SAMN15208332, SAMN15208333, SAMN15208334, SAMN15208335, SAMN15208336, SAMN15208337, SAMN15208338, SAMN15208339, SAMN15208340, SAMN15208341, SAMN15208342, SAMN15208343, SAMN15208344 and SAMN15208345.

## Figures and Tables

**Table 1:** Clinical characteristics of Spanish patients.

		PHV	No PHV	*p*
		(*n* = 15)	(*n* = 85)	
	Gender Male/Female	66.7% (10)/33.3% (5)	55.3% (47)/44.7% (38)	0.412
	Age (years)	51.2 ± 16.2	48.8 ±15.3	0.668
	Type of dialysis- Automated PD	80% (12)	71.8% (61)	0.508
	Time on PD (months)	32.6 ± 21.9	21.7±14	0.076
**Medical record**	Diabetes	6.7% (1)	9.4% (8)	0.732
	Hypertension	93.3% (14)	83.5% (71)	0.327
	Ischemic cardiopathy	13.3% (2)	7.1% (6)	0.409
	Stroke	13.3% (2)	11.8% (10)	0.863
	Peripheral artery disease	13.3% (2)	16.5% (14)	0.76
**Pharmacological treatment**	ACEI	26.7% (4)	57.6% (49)	0.027
	ARB	40% (6)	31.8% (27)	0.532
	B-blockers	53.3% (8)	47.1% (40)	0.654
	Steroids	20% (3)	14.1% (12)	0.556
	Statins	20% (3)	51.2% (43)	0.026
	Antiplatelets	6.7% (1)	9.5% (8)	0.723
	Anticoagulants	6.7% (1)	2.4% (2)	0.372
**Other PD-related data**	Biocompatible dialysis solutions use	20% (3)	56.5% (48)	0.009
	Previous peritonitis episodes	33.3% (5)	36.5% (31)	0.815
	Accumulated days of peritonitis	9.7 ± 11.3	4.6 ± 3.3	0.569
	Residual renal function (ml/min/1.73m^2^)	2.7±2.1	4.8 ± 3.1	0.001

**Table 2:** microRNAs (miRNAs) potentially regulated in PHV as compared to no PHV by a differential expression analysis (Cufflinks).

Cluster ID	Predicted Targets in Mirbase	Chr	Region Start	Region End	log2 (Fold_Change)	*p*-Value	*q*-Value
hsa-mir-1185-1-3p	2269	chr14	101042976	101043062	3.05	7.55 × 10^−3^	4.15 × 10^−2^
hsa-mir-1193	no	chr14	101030051	101030129	-inf	5.00 × 10^−4^	5.10 × 10^−3^
hsa-mir-1246	no	chr2	176600979	176601052	−1.64	7.50 × 10^−4^	7.07 × 10^−3^
hsa-mir-1299	5405	chr9	40929009	40929092	−1.49	8.25 × 10^−3^	4.43 × 10^−2^
hsa-miR-154-3p	83	chr14	101059754	101059838	2.47	5.00 × 10^−5^	7.77 × 10^−4^
hsa-miR-154-5p	165	chr14	101059754	101059838	2.47	5.00 × 10^−5^	7.77 × 10^−4^
hsa-miR-200a-3p	110	chr1	1167862	1167952	2.13	2.45 × 10^−3^	1.82 × 10^−2^
hsa-miR-323b-5p	998	chr14	101056218	101056300	4.24	1.50 × 10^−4^	1.91 × 10^−3^
hsa-miR-34a-5p	319	chr11	111513438	111513515	1.70	0.0072	4.03 × 10^−2^
hsa-miR-34c-5p	319	chr11	111513438	111513515	1.70	7.20 × 10^−3^	4.03 × 10^−2^
hsa-miR-369-5p	294	chr14	101065597	1010656,67	2.35	6.50 × 10^−3^	3.78 × 10^−2^
hsa-mir-376a-2-5p	no	chr14	101040068	101040148	-inf	5.00 × 10^−5^	7.77 × 10^−4^
hsa-miR-377-3p	635	chr14	101062049	101062118	3.20	5.00 × 10^−5^	7.77 × 10^−4^
hsa-miR-383-5p.1	235	chr8	14853437	14853510	−2.50	6.45 × 10^−3^	3.76 × 10^−2^
hsa-miR-383-5p.2	223	chr8	14853437	14853510	−2.50	6.45 × 10^−3^	3.76 × 10^−2^
hsa-miR-412-3p	2888	chr14	101065446	101065537	2.70	6.50 × 10^−3^	3.78 × 10^−2^
hsa-miR-494-5p	712	chr14	101029633	101029714	4.03	5.00 × 10^−5^	7.77 × 10^−4^
hsa-mir-542-5p	1047	chrX	134541340	134541437	3.21	5.00 × 10^−5^	7.77 × 10^−4^
hsa-mir-548ad-3p	1717	chr2	35471404	35471486	-inf	8.70 × 10^−3^	4.60 × 10^−2^
hsa-miR-6507-3p	no	chr10	98924498	98924568	-inf	5.00 × 10^−5^	7.77 × 10^−4^
hsa-miR-6507-5p	no	chr10	98924498	98924568	-inf	5.00× 10^−5^	7.77 × 10^−4^
hsa-miR-651-5p	3719	chrX	8126964	8127061	2.12	8.30× 10^−3^	4.44 × 10^−2^
hsa-mir-7641	no	chr11	10425259	104252651	1.60	5.55 × 10^−3^	3.37 × 10^−2^
hsa-mir-873-3p	4301	chr9	28888878	28888955	−2.58	7.50 × 10^−4^	7.07 × 10^−3^

## Supplementary Materials

Supplementary Materials can be found at https://www.mdpi.com/1422-0067/21/16/5824/s1.
