# Supplementary material for: Increased miR-7641 Levels in Peritoneal Hyalinizing Vasculopathy in Long-Term Peritoneal Dialysis Patients
Source: Int J Mol Sci. 2020 Aug 13;21(16):5824. doi: 10.3390/ijms21165824 (PMC7461593; doi:10.3390/ijms21165824)
Supplement: Supplementary file 1 [file ijms-21-05824-s001.pdf]

International Journal of
Molecular Sciences

Increased miR-7641 Levels in Peritoneal Hyalinizing Vasculopathy in Long-Term Peritoneal Dialysis Patients

Raquel Díaz, Pilar Sandoval, Raul R. Rodrigues-Diez, Gloria del Peso
José A Jiménez-Heffernan, Ricardo Ramos-Ruíz, Carlos Llorens, Gustavo Laham,
Mabel Alvarez-Quiroga, Manuel López-Cabrera, Marta Ruiz-Ortega, María A. Bajo and Rafael Selgas

Table of Contents

Name	Title
Figure S1	A) Immunofluorescence staining for CD31 shows lower expression in a PHV as compared to a control vessel. B) Left, FSP-1 negative endothelial cells in a control vessel. Right, FSP-1 positive endothelial cells in a PHV.
Table S1	Summary of sequencing data
Table S2	microRNAs selected as candidates for validation studies
Table S3	deltaCt values (expressed in arbitrary units) for miR-7641

Figure S1

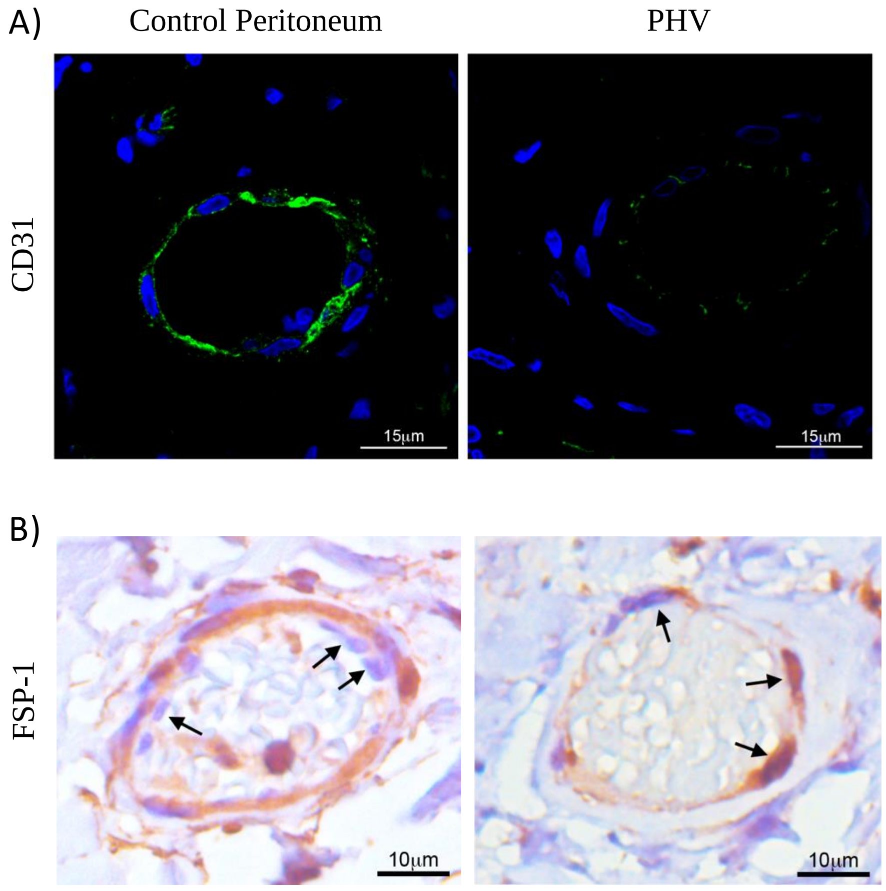

Table S1

Sample	Reads pre-Processing (k)	Reads After Processing (K)	% Filtered Reads	% mapped vs Human Genome	Condition	Sample2
RR1	16465,2	12324,3	74,85%	94,1%	Vasculopathy	RR1
RR2	16131,4	15005,0	93%	89,1%	Vasculopathy	RR2
RR3	14871,6	13081,7	87,96%	83,0%	Vasculopathy	RR3
RR4	18351,1	14363,4	78,27%	94,9%	Vasculopathy	RR4
RR5	15975,1	12616,5	78,98%	96,0%	Vasculopathy	RR5
RR6	24299,3	20752,9	85,41%	73,65	Vasculopathy	RR6
RR7	17167,1	15088,8	87,89%	35%**	Vasculopathy	RR7
RR9	19705,8	19196,9	97,42%	9,6%**	Vasculopathy	RR9
RR10	20748,0	19943,6	96,12%	17,45%**	Vasculopathy	RR10
RR11	25115,1	24699,8	98,35%	10,64%**	Vasculopathy	RR11
RR12	24158,3	21044,0	87,11%	70,4%	Control	RR12
RR13	20403,1	19135,8	93,79%	87,4%	Control	RR13
RR14	11569,9	8977,4	77,59%	55,1%	Control	RR14
RR15	20418,7	17599,1	86,19%	33,13%**	Control	RR15
RR17	23466,7	22335,3	95,25%	9,86%**	Control	RR17
RR18	25661,8	25408,5	99,01%	6,17%**	Control	RR18
RR19	9243,7	6461,2	69,90%	21,85%**	Control	RR19
RR20	16214,7	14601,5	90,05%	78,9%	Control	RR20
RR21	19394,5	17824,2	91,90%	91,8%	Vasculopathy	RR21

Table S2

1	hsa-mir-376a-2-5p	targets mature miRNA: hsa-miR-376a-2-5p,	5'GGUAGAUUUUCCUUCUAUGGU
2	hsa-mir-548ad-3p	targets mature miRNA: hsa-miR-548ad-3p,	5'GAAAACGACAAUGACUUUUGCA
3	hsa-miR-323b-5p	targets mature miRNA: hsa-miR-323b-5p, bta-miR-453, mml-miR-323b-5p, mml-miR-453, ppy-miR-453, ptr-miR-453	5'AGGUUGUCCGUGGUGAGUUCGCA
4	hsa-miR-494-5p	targets mature miRNA: hsa-miR-494-5p,	5'AGGUUGUCCGUGUUGUCUUCUCU
5	hsa-mir-542-5p	targets mature miRNA: hsa-miR-542-5p, mml-miR-542-5p, ppy-miR-542-5p	5'UCGGGGAUCAUCAUGUCACGAGA
6	hsa-miR-377-3p	targets mature miRNA: hsa-miR-377-3p, bta-miR-377, eca-miR-377, mml-miR-377-3p, mmu-miR-377-3p, oar-miR-377-3p, ppy-miR-377, ptr-miR-377	5'AUCACACAAAGGCAACUUUUGU
7	hsa-mir-1185-1-3p	targets mature miRNA: hsa-miR-1185-1-3p,	5'AUAUACAGGGGGAGACUCUUAU
8	hsa-miR-412-3p	targets mature miRNA: hsa-miR-412-3p, mml-miR-412-3p, ppy-miR-412, ptr-miR-412	5'ACUUCACCUGGUCCACUAGCCGU
9	hsa-miR-154-5p	targets mature miRNA: hsa-miR-154-5p, cgr-miR-154-5p, ggo-miR-154, mml-miR-154-5p, mmu-miR-154-5p, mne-miR-154, ppa-miR-154, ppy-miR-154, ptr-miR-154, rno-miR-154-5p	5'UAGGUUAUCCGUGUUGCCUUCG
10	hsa-miR-154-3p	targets mature miRNA: hsa-miR-154-3p, cgr-miR-154-3p, mml-miR-154-3p, mmu-miR-154-3p, rno-miR-154-3p	5'AAUCAUACACGGUUGACCUAUU
11	hsa-miR-369-5p	targets mature miRNA: hsa-miR-369-5p, cgr-miR-369-5p, mml-miR- 5p, mmu-miR-369-5p, ppy-miR-369-5p, rno-miR-369-5p	5'AGAUCGACCGUGUUAUAUUCGC 369-
12	hsa-miR-200a-3p	targets mature miRNA: hsa-miR-200a-3p, eca-miR-200a, fru-miR-200a, gga-miR-200a-3p, ggo-miR-200a, mdo-miR-200a-3p, mml-miR-200a-3p, mmu-miR-200a-3p, ppy-miR-200a, ptr-miR-200a, rno-miR-200a-3p, tni-miR-200a, xtr-miR-200a	5'UAACACUGUCUGGUAACGAUGU
13	hsa-miR-651-5p	targets mature miRNA: hsa-miR-651-5p, ggo-miR-651	5'UUUAGGAUAAGCUUGACUUUUG
14	hsa-miR-34C-5p	targets mature miRNA: hsa-miR-34c-5p, cfa-miR-34c, cgr-miR-34c-5p, eca-miR-34c, gga-miR-34c-5p, mdo-miR-34c-5p, mml-miR-34c-5p, mmu-miR-34c-5p, oan-miR-34a-5p, ppy-miR-34c-5p, rno-miR-34c-5p, ssc-miR-34c, tgu-miR-34b	5'AGGCAGUGUAGUUAGCUGAUUGC
15	hsa-miR-34a-5p	targets mature miRNA: hsa-miR-34a-5p, age-miR-34a, bta-miR-34a, ccr-miR-34, cfa-miR-34a, cgr-miR-34a, dre-miR-34a, eca-miR-34a, ggo-miR-34a, ipu-miR-34a, lla-miR-34a, mml-miR-34a-5p, mmu-miR-34a-5p, mne-miR-34a, ppa-miR-34a, ppy-miR-34a, ptr-miR-34a, rno-miR-34a-5p, sla-miR-34a, ssc-miR-34a, tgu-miR-34a	5'UGGCAGUGUCUUAGCUGGUUGU
16	hsa-mir-7641	targets mature miRNA: hsa-miR-7641,	5'UUGAUCUCGGAAGCUAAGC
17	hsa-mir-873-3p	targets mature miRNA: hsa-miR-873-3p,	5'GGAGACUGAUGAGUUCCCGGGA
18	hsa-miR-383-5p.1	targets mature miRNA: hsa-miR-383-5p, bta-miR-383, cfa-miR-383, eca-miR-383, gga-miR-383-5p, mdo-miR-383-5p, mml-miR-383, ppy-miR-383, ptr-miR-383, tgu-miR-383-5p, xtr-miR-383	5'AGAUCAGAAGGUGAUUGUGGCU
19	hsa-mir-1193	targets mature miRNA: hsa-miR-1193, ggo-miR-1193, oar-miR-1193-5p, ppy-miR-1193	5'GGGAUGGUAGACCGGUGACGUGC
20	hsa-mir-1246	targets mature miRNA: hsa-miR-1246, bta-miR-1246, ptr-miR-1246	5'AAUGGAUUUUUGGAGCAGG
21	hsa-mir-1299	targets mature miRNA: hsa-miR-1299,	5'UUCUGGAAUUCUGUGUGAGGGA
22	hsa-miR-6507-5p	targets mature miRNA: hsa-miR-6507-5p,	5'GAAGAAUAGGAGGGACUUUGU
23	hsa-miR-134-5p	targets mature miRNA: hsa-miR-23a-3p, age-miR-23a, ccr-miR- 23a, cgr-miR-23a-3p, eca-miR-23a, ggo-miR-23a, ipu-miR-23a, lca-miR-23a, mml-miR-23a-3p, mmu-miR-23a-3p, mne-miR-23a, ppa-miR-23a, ppy-miR-23a, ptr-miR-23a, rno-miR-23a-3p, sla-miR- 23a, ssc-miR-23a, xla-miR-23a, xtr-miR-23a	5'AUCACAUUGCCAGGGAUUUCC
24	hsa-miR-23a-3p	targets mature miRNA: hsa-miR-134-5p, cfa-miR-134, cgr-miR-134, eca-miR-134, mml-miR-134-5p, mmu-miR-134-5p, ptr-miR-134, rno-miR-134-5p	5'UGUGACUGGUUGACCAGAGGGG
25	hsa-miR-132-3p	targets mature miRNA: hsa-miR-132-3p, bta-miR-132, ccr-miR-132a, cgr-miR-132-3p, dre-miR-132-3p, eca-miR-132, fru-miR-132, ipu-miR-132b, mdo-miR-132-3p, mml-miR-132-3p, mmu-miR-132-3p, ppy-miR-132, rno-miR-132-3p, ssc-miR-132, tgu-miR-132, tni-miR-132, xtr-miR-132	5'UAACAGUCUACAGCCAUGGUCG
26	hsa-miR-203b-5p	targets mature miRNA: hsa-miR-203b-5p,	5'UAGUGGUCCUAAACAUUUCACA

Table S3

Sample Name	Detector	DCt	Mask	Task	Pathology	Grade	SampleName	Factor (Index)[Grade]	2E-dCt
Healthy.1	7641	4,5075	valid	Target	Healthy	Healthy	SANOS.1	4	0,04396502
healthy.2	7641	1,135	valid	Target	Healthy	Healthy	SANOS.2	4	0,45533492
healthy.3	7641	1,82	valid	Target	Healthy	Healthy	SANOS.3	4	0,28322097
healthy.4	7641	3,695	valid	Target	Healthy	Healthy	SANOS.4	4	0,07721366
healthy.5	7641	2	valid	Target	Healthy	Healthy	SANOS.5	4	0,25
RR12	7641	1,83	valid	Target	NO.VH	V0	RR12	0	0,28126462
RR14	7641	1,7225	valid	Target	NO.VH	V0	RR14	0	0,30302317
VASC.CONTROL.1	7641	3,8	valid	Target	NO.VH	V0	VASCU.CONTROL.1	0	0,07179365
VASC.CONTROL.2	7641	4,18	valid	Target	NO.VH	V0	VASCU.CONTROL.2	0	0,05516894
VASC.CONTROL.3	7641	5,1	valid	Target	NO.VH	V0	VASCU.CONTROL.3	0	0,02915728
X1.357095	7641	-1,09	valid	Target	NO.VH	V0	X1.357095	0	2,12874036
X13.438604	7641	0,36	valid	Target	NO.VH	V0	X13.438604	0	0,77916458
X24.446973	7641	-0,615	valid	Target	NO.VH	V0	X24.446973	0	1,531558
X9.444811	7641	-0,25	valid	Target	NO.VH	V0	X9.444811	0	1,18920712
RR1	7641	0,5025	valid	Target	VH	V1	RR1	1	0,70588252
RR4	7641	1,22	valid	Target	VH	V1	RR4	1	0,42928272
RR7	7641	1,6675	valid	Target	VH	V1	RR7	1	0,31479838
VASC.1	7641	2,92	valid	Target	VH	V1	VASCU.1	1	0,13212726
VASC.2	7641	-0,105	valid	Target	VH	V1	VASCU.2	1	1,07549439
X18.446063	7641	-0,79	valid	Target	VH	V1	X18.446063	1	1,72907446
X30.452507	7641	0,665	valid	Target	VH	V1	X30.452507	1	0,6306887
X16.387195	7641	-0,785	valid	Target	VH	V1	X16.387195	1	1,72309232
X11.343537	7641	-1,06	valid	Target	VH	V3	X11.343537	2	2,08493152
X25.456285	7641	-0,975	valid	Target	VH	V3	X25.456285	2	1,9656412
X28.367621	7641	-0,78	valid	Target	VH	V3	X28.367621	2	1,71713087
